# Lipid metabolism patterns and relevant clinical and molecular features of coronary artery disease patients: an integrated bioinformatic analysis

**DOI:** 10.1186/s12944-022-01696-w

**Published:** 2022-09-10

**Authors:** Yanhui Liao, Zhenzhen Dong, Hanhui Liao, Yang Chen, Longlong Hu, Zuozhong Yu, Yi Xia, Yuanbin Zhao, Kunpeng Fan, Jingwen Ding, Xiongda Yao, Tianhua Deng, Renqiang Yang

**Affiliations:** 1 Department of Cardiovascular Medicine, The Second Affiliated Hospital of Nanchang University, Nanchang, Jiangxi 330006 People’s Republic of China; 2 Department of Emergency, The Second Affiliated Hospital of Nanchang University, Nanchang, Jiangxi 330006 People’s Republic of China

**Keywords:** Coronary artery disease (CAD), Lipid metabolism, Classification, Bioinformatics

## Abstract

**Background:**

Hyperlipidaemia is an important factor that induces coronary artery disease (CAD). This study aimed to explore the lipid metabolism patterns and relevant clinical and molecular features of coronary artery disease patients.

**Methods:**

In the current study, datasets were fetched from the Gene Expression Omnibus (GEO) database and nonnegative matrix factorization clustering was used to establish a new CAD classification based on the gene expression profile of lipid metabolism genes. In addition, this study carried out bioinformatics analysis to explore intrinsic biological and clinical characteristics of the subgroups.

**Results:**

Data for a total of 615 samples were extracted from the Gene Expression Omnibus database and were associated with clinical information. Then, this study used nonnegative matrix factorization clustering for RNA sequencing data of 581 lipid metabolism relevant genes, and the 296 patients with CAD were classified into three subgroups (NMF1, NMF2, and NMF3). Subjects in subgroup NMF2 tended to have an increased severity of CAD. The CAD index and age of group NMF1 were similar to those of group NMF3, but their intrinsic biological characteristics exhibited significant differences. In addition, weighted gene coexpression network analysis (WGCNA) was used to determine the most important modules and screen lipid metabolism related genes, followed by further analysis of the DEGs in which the significant genes were identified based on clinical information. The progression of coronary atherosclerosis may be influenced by genes such as PTGDS and DGKE.

**Conclusion:**

Different CAD subgroups have their own intrinsic biological characteristics, indicating that more personalized treatment should be provided to patients in each subgroup, and some lipid metabolism related genes (PDGTS, DGKE and so on) were related significantly with clinical characteristics.

## Introduction

Coronary artery disease (CAD) is a major cause of both death and disability worldwide and is responsible for more than one-third of all deaths in individuals over the age of 35 [1–3]. CAD is an ischaemic heart disease caused by lipid deposition in the blood vessel wall and the formation of atherosclerotic plaque, which leads to lumen stenosis and reduction in the myocardial blood supply. Once the plaque is ruptured, it quickly blocks the blood vessels, resulting in a severe shortage of blood supply to the myocardial tissue and then to myocardial ischaemic necrosis, resulting in a series of secondary lesions, the most serious of which is acute myocardial infarction [4].

As with most complex diseases, the risk of CAD is affected by the interaction of genetic and lifestyle factors. It has been reported that more than half of the risk of CAD is associated with dyslipidaemia, and the increase in major blood lipid indicators is closely related to the occurrence of cardiovascular risk events [5]. Lipid metabolism pathways might be an indicator of increased severity. From genome‐wide association studies (GWASs), several genetic variants have been found to be closely related to CAD [6, 7], and CAD is highly heritable with genetic risk accounting for 40% to 60% of the susceptibility to CAD [8, 9]. However, our ability to understand the molecular basis of CAD remains limited [8]. In this regard, it is necessary to address the association of lipid metabolism pathways in candidate genomes with CAD development.

Unlike previous the most studies, which only paid attention to the differences between normal controls and CAD cases, this study mainly focused on the differentially expressed genes between CAD cases. This will help to reveal the heterogeneity between patients and predict clinical endpoints. It will also contribute to the management of patients. CAD patients were classified according to lipid metabolism related genes to deepen the understanding of the molecular mechanism of CAD, and determine the key genes and associated signalling pathways related to CAD, which could play essential roles in gene therapy.

## Materials and methods

### Data collection

Two datasets (GSE1228 [10] and GSE20686 [11]) were fetched from the Gene Expression Omnibus (GEO) database (https://www.ncbi.nlm.nih.gov/geo/). GSE12288, including 222 samples from 112 healthy controls and 110 CAD cases, provided the Duke CAD index(CADi) [12] of CAD patients, which is an assessment of the severity of CAD. In addition, GSE20686 was composed of GSE20680 and GSE20681, which involved 195 and 198 samples, respectively. This study used the R/Bioconductor package GEO query [13] to extract GEO objects. The GEO objects, including the gene expression matrixes and clinical information, were returned by the getGEO function.

### Removal of batch effects

To obtain a larger cohort of CAD cases and compare samples among different cohorts, it is necessary to correct the batch effects among the three objects firstly. The “sva” package [14, 15] in R is often used to identify, estimate and remove the variation produced in high-throughput gene expression microarray experiments to eliminate batch effects. In this study, in the R language environment, Using the "ComBat" function in the "SVA" package, batch effects were processed. After batch correction was completed, principal component analysis (PCA) was performed to assess whether the batch effects were eliminated.

### Differentially expressed genes (DEGs) screening

In this study, 581 lipid metabolism related genes (LMRGs) were downloaded from the Molecular Signatures Database website (https://www.gsea-msigdb.org/gsea/msigdb/index.jsp).The advantage of linear models for microarray data (limma) is that the linear model is used to analyse the experiment as a whole, and researchers can adjust multiple influencing factors or individual factors. The linear model can study the dependence between variables by using multiple variables as covariates, and the adoption of linear models for analysing differential gene expression [16]. A limma package in R was used to screen DEGs between CAD cases and normal samples based on LMRGs and this study considered |log2fold change (FC)|> 1 and Franklin Delano Roosevelt value < 0.05 as the thresholds for selecting the significant DEGs [16].

### Functional enrichment analysis

Metascape (http://metascape.Org), a free analysis and gene annotation resource that helps biologists understand one or multiple gene lists, integrates many authoritative databases such as DrugBank, Gene Ontology (GO), UniProt and Kyoto Encyclopedia of Genes and Genomes (KEGG), which not only allows complete biological function annotation and enrichment analysis, but also facilitates protein network analysis and drug reaction analysis. At the same time, the data of the platform is updated very frequently, which ensures the reliability and relevance of the data in the database [17]. Terms with ≥ 3 enriched genes and a *P* value < 0.01 were defined as significant and classified into clusters according to their similar membership degree. The most enriched term within a cluster was chosen as the one to represent the cluster.

### PPI (protein–protein interactions) network construction

The Search Tool for the Retrieval of Interacting Genes (STRING) database (version 11.0; www.string-db.org) [18], the most commonly used online tool for PPI network analysis in the biomedical field, was used to develop the PPI network of DEGs. Moreover, the interaction score was defined as more than 0.4. Finally, the PPI network was visualized by using the Cytoscape software [19].

### Identification of CAD subgroups

A previously reported list of 581 LMRGs was prepared for nonnegative matrix factorization (NMF) clustering [20]. The NMF method extracts the biological correlation coefficient of the data in the gene expression matrix, organizes the genes and samples, grasps the internal structural characteristics of the data, and then groups the samples, and this method is widely used in disease typing. Unsupervised NMF clustering was achieved through the R package “NMF” on the meta-dataset [21]. The k values at which the cophenetic curve drops fastest were selected as the optimal number of clusters [22].

### Clinical characterization of the three subgroups

A pairwise proportion test was used to compare the proportion of males in the subgroups. In addition, the differences in age and CADi between the subclasses were tested by pairwise Wilcoxon’s rank‐sumtest.

### Specific upregulated genes in each subgroup

By comparing the patients in a specific subgroup with those in every other subgroup, subgroup-specific up-regulated genes were identified. This used Wilcoxon’s‐sum rank test to analyse differential expression with the thresholds absolute difference of means > 0.2 and Benjamini‐Hochberg adjusted *p* < 0.05.

### Using WGCNA to screen disease-related modules and genes

In order to assess lipid metabolism related gene expression, weighted gene coexpression network analysis (WGCNA) was implemented. WGCNA is a well-developed and widely used systematic biological algorithm [23]. It constructs a scale-free network on the basis of the relation matrix and constructs the topological overlap matrix. Then, according to the degree of topological difference, the genes with strong coexpression relationships were divided into different gene modules, which were investigated as a whole, and trait (phenotypic) information was further introduced to explore the correlation between the eigengenes of each module and the disease status, Finally, enrichment analysis, core gene screening and other methods to determine the key signalling pathways or key genes related to traits [24].

### DEGs screening based on genes in modules

After selecting the module with the highest correlation with the disease, this study analysed the DEGs within the module further. This study calculated the *P* value of genes and the adjusted *P* value, which were used by the t test method and Benjamini and Hochberg's method, respectively. An adjusted *P*-value < 0 0.05 between two groups was set as the selection criterion to screen out DEGs.

## Results

### Characteristics of coronary artery disease subjects

A total of 615 samples were included in this research, and according to the degree of coronary artery stenosis, the patients were divided into a control group(luminal stenosis of < 50%) and a case group(≥ 50% stenosis in ≥ 1 major vessel) (Table 1).

**Table 1:** Cohorts and sample information included in this research

Cohort	Control	Case	Total
GSE12288	112	110	222
GSE20680	108	87	195
GSE20681	99	99	198
Total	319	296	615

Cases: Patients with ≥ 50% stenosis in ≥ 1 major vessel;

Controls: Patients with luminal stenosis of < 50%

### Removal of the batch effect

**Fig. 1:**
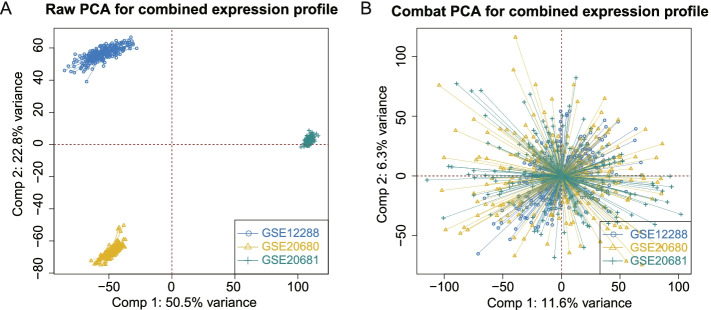
Principal component analysis (PCA) shows the degree of similarity between samples through cluster analysis. The closer the spatial distance of different samples was, the smaller the difference between samples was. **A** Before batch-effect removal. **B** After batch-effect removal

To eliminate batch effects between the different datasets, the ComBat method was applied. In the principal component analysis diagram before the cross‐platform standardization, the sample scores of the three batches were distinguished (Fig. 1A). In contrast, after batch effect correction, the samples of the three batches were mixed together (Fig. 1B), indicating that the batch effect was eliminated.

### Identification of DEGs and functional enrichment analysis

**Fig. 2:**
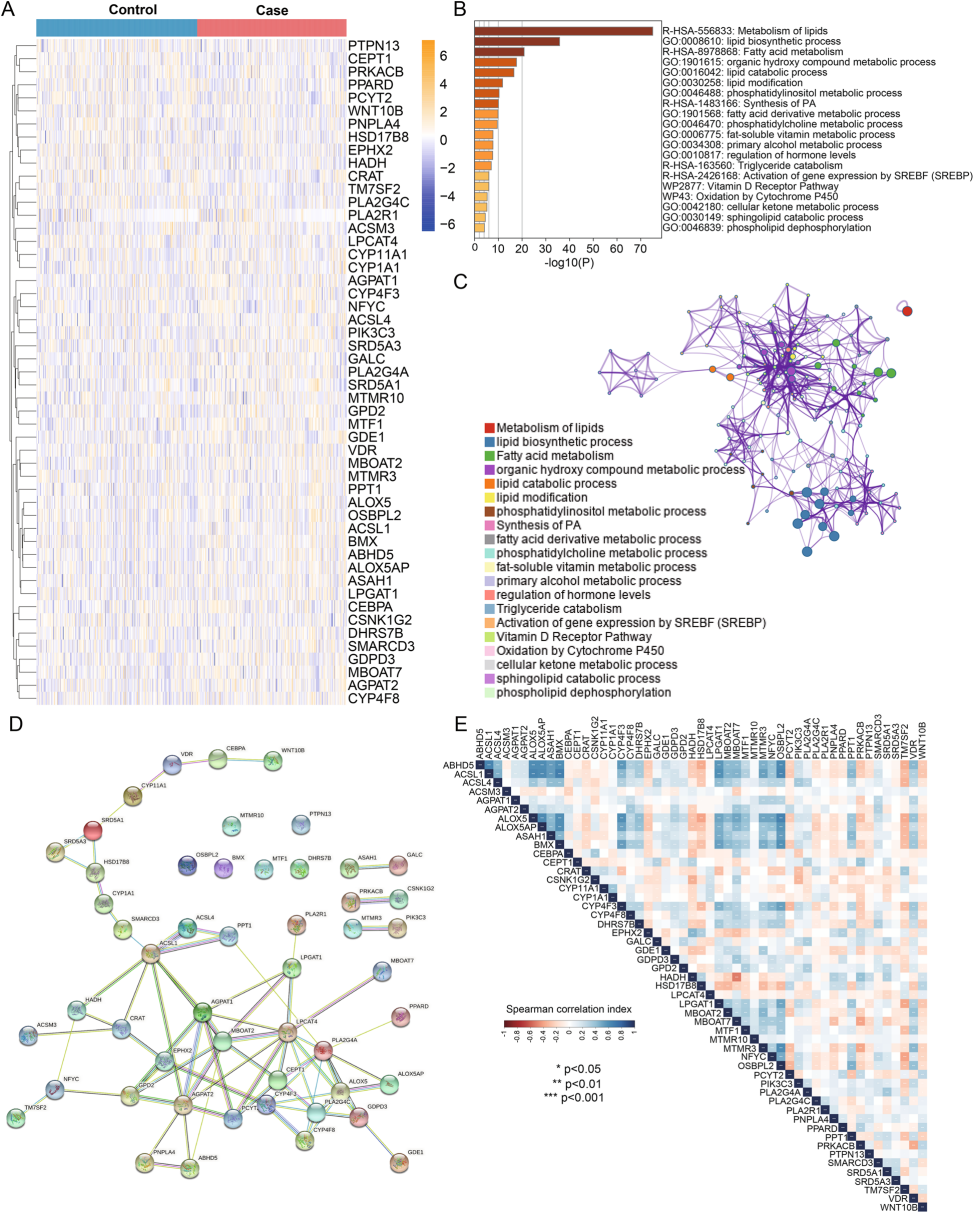
Identification of differentially expressed genes (DEGs), functional enrichment analysis and protein‒protein interaction (PPI) network of DEGs. **A** The distinguishable mRNA expression between CAD samples and healthy controls is displayed by heatmap visualization. **B** Coloured by *P* values, heatmap of enriched terms across input gene lists. **C** Coloured by cluster ID, where nodes that have the same cluster ID are relatively close to each other. **D** Protein‒protein interaction network of 51 differentially expressed LMRGs. **E** Correlation heatmap showing the coexpression patterns of the 51 differentially expressed LMRGs in CAD cases

DEGs were identified using the R package “limma”. The distinguishable LMRG mRNA expression between CAD samples and healthy controls is displayed by heatmap visualization in Fig. 2A, including 51 DEGs. As shown in Fig. 2B, C, the KEGG pathway of the genes was found to be mainly enriched in “metabolism of lipids”, “fatty acid metabolism”, “ynthesis of PA”, “triglyceride catabolism” and “activation of gene expression by SREBF(SREBP)”. Additionally, GO analysis showed that the genes were mainly enriched in “lipid biosynthetic process”, “organic hydroxy compound metabolic process”, ” lipid catabolic process”, ”lipid modification”, ”phosphatidylinositol metabolic process” and so on.

### PPI network

As a result of removing the isolated nodes, the PPI network consisted of 74 edges and 51 nodes (Fig. 2D). Several genes were identified as hub genes in the PPI network, including ACSL1, AGPAT1, AGPAT2, ALOX5, CEPT1 and LPCAT4. In the network, there were 8 nodes connected to AGPAT1, which had the highest degree of connectivity.

### NMF identifies three subclasses in CAD

**Fig. 3:**
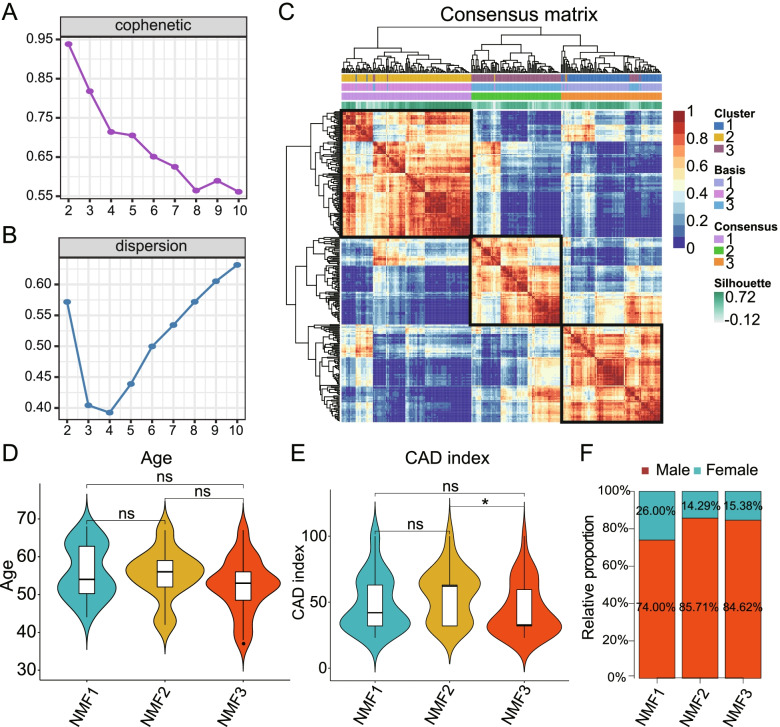
Identification of CAD subgroups using NMF consensus clustering and the comparison of clinical features between the subgroups. **A**, **B** NMF clustering using 581 lipid metabolism related genes. Cophenetic and dispersion correlation coefficients for k = 2–5 are shown. **C** The heatmap shows the consensus matrix with a cluster count of 3. When k = 3, the heatmap maintains clear boundaries, indicating stable clustering for the cases. **D** Age of each subgroup. **E** CAD index of each subgroup. **F** Proportion of males in each subgroup

The 581 previously reported LMRGs were selected as the foundation of NMF analysis. The meta-dataset comprising 296 CAD samples from GSE12288, GSE20680 and GSE20681 was classified based on the expression profile of LMRGs using NMF consensus clustering. The optimal value of k was determined by calculating the cophenetic correlation coefficient (Fig. 3A), and after comprehensive consideration, the optimal number of clusters was selected as k = 3. The heatmap still maintained a clear boundary, indicating that the sample had stable and robust clustering (Fig. 3C).Three distinct molecular subclasses were designated NMF1, NMF2, and NMF3, with 105, 97, and 94 cases respectively (Table 2), which had different gene expression patterns.

**Table 2:** Number of cases in each subgroup

Subgroup	NMF1	NMF2	NMF3	Total
GSE12288	50	21	39	110
GSE20680	24	37	26	87
GSE20681	31	39	29	99
Total	105	97	94	296

To describe the clinical features of the subgroups, the age, CADi and sex were studied for CAD cases of the GSE12288 dataset. In terms of age, there was no significant difference between the groups. Moreover, As compared to subgroups NMF2 and NMF3, subgroup NMF1 had a lower proportion of males. In addition, subgroup NMF2 had a higher CADi than subgroups NMF3. However, no significant differences were found between subgroups NMF1 and NMF3 in CADi. The CADi of the NMF2 group seemed to be higher than that of the NMF1 group, but the difference was not significant, which demonstrated that cases in NMF2 subgroup may have had more severe CAD.

**Fig. 4:**
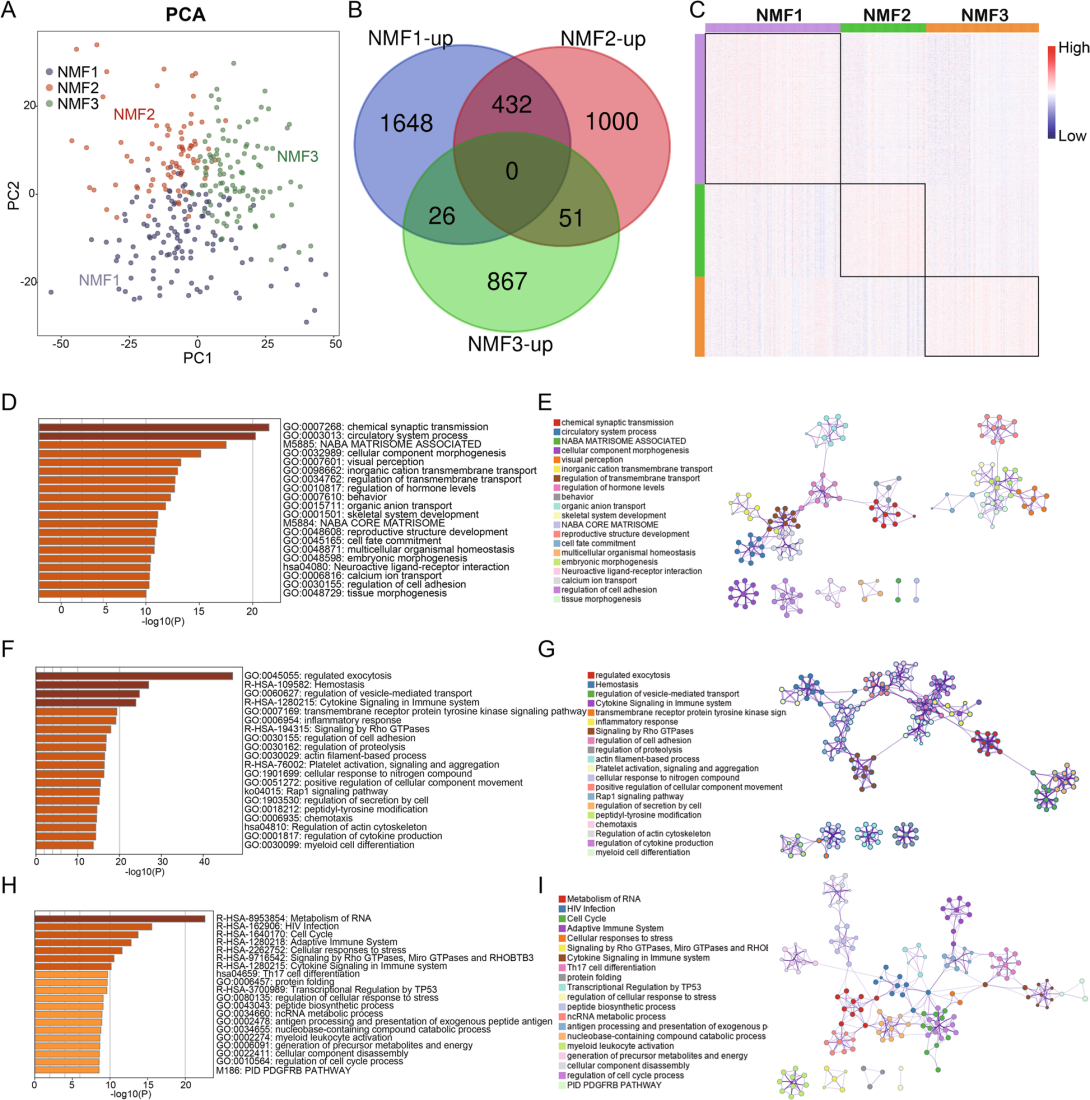
Principal component analysis (PCA) and the expression patterns of upregulated genes specific to subgroups. **A** PCA supported the classification into three CAD subgroups. **B** Venn diagram of upregulated DEGs from the intersection of the three subgroups. **C** Heatmap visualization of specific upregulated genes in each CAD subgroups. **D**, **F**, **H** Coloured by *P* values, heatmap of enriched terms across input gene lists of NMF1, NMF2, and NMF3, respectively. **E**, **G** and **I** are coloured by cluster ID, where nodes that have the same cluster ID are generally close to each other

To confirm the subgroup assignments, PCA was performed to reduce the dimension of traits, and the subgroup designations were in accordance with the consensus matrix heatmap (Fig. 4A), indicating that the three different molecular subgroups of CAD had distinct gene expression patterns. To analyse all the differentially expressed genes, By using the limma package, specific upregulated genes were identified, as shown in the Venn diagram, and 1648, 1000, and 867 genes were specifically upregulated in subgroups NMF1, NMF2, and NMF3, respectively (Fig. 4B, C). As shown in Fig. 4D-I, enrichment analysis of specific up-regulated genes was performed in each group. Genes specifically upregulated in the NMF1 subgroups were mainly enriched in “chemical synaptic transmission”, “circulatory system process”, “NABA matrisome associated”, “cellular component morphogenesis” and “visual perception”. Genes specifically upregulated in subgroups NMF2 were significantly enriched in “regulated exocytosis”, haemostasis”, “regulation of vesicle-mediated transport”, “cytokine signalling in immune system”, “transmembrane receptor protein tyrosine kinase signalling pathway”. Genes specifically upregulated in subgroup NMF1 were found to be enriched in “metabolism of RNA”, “HIV infection”, “cell cycle”, “adaptive immune system” and “cellular responses to stress”.

### WGCNA modules and module-trait analysis

**Fig. 5:**
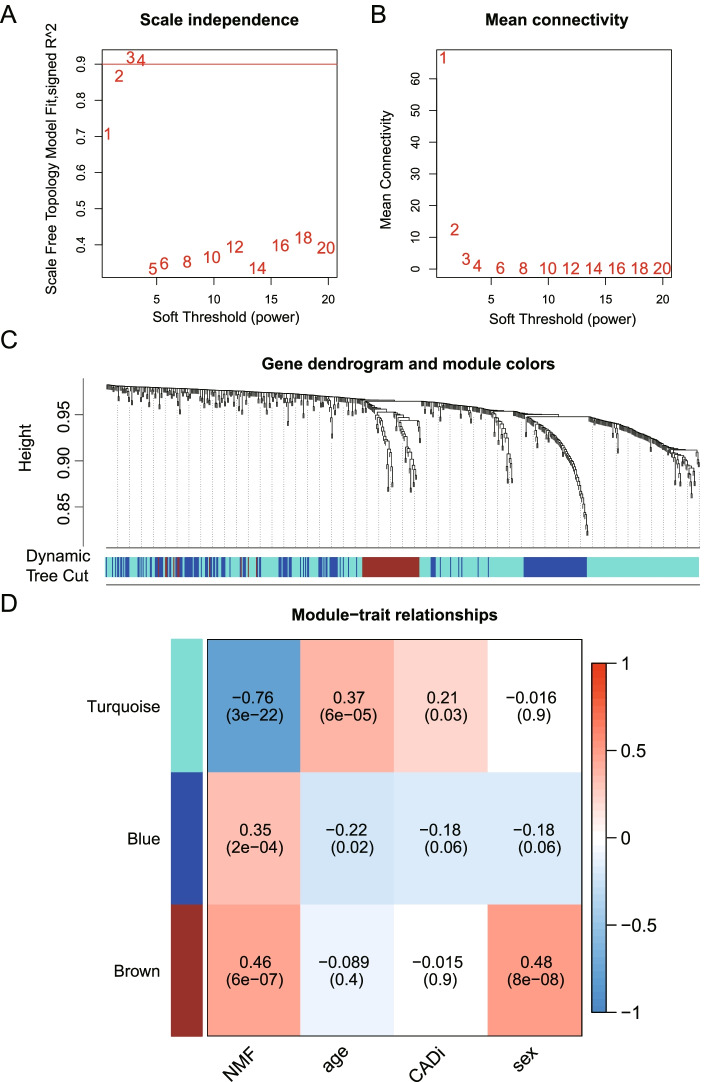
Weighted gene coexpression network analysis. The correlation coefficient (R2) (**A**) and mean connectivity (**B**) scatter diagram corresponding to different *P* values. **C** Module eigengene dendrogram. The vertical axis represents the degree of topological difference between genes, and the farther the distance on the vertical axis, the greater the degree of topological difference between genes, meaning the weaker the coexpression relationship; the horizontal axis represents different modules, and the colour represents a module. **D** The module-character correlation heatmap between the modules and four clinical traits. Four distinct qualities are represented by the horizontal axis, while various gene coexpression modules are shown by the vertical axis. The colour in each cell in the graph indicates the correlation between the corresponding module and the character. Darker shades of red indicate stronger positive correlations. Deeper shades of green indicate stronger negative correlations. A correlation number is shown in each cell, and the significance of the correlation(p) is shown in parentheses below

According to the standard of the scale-free network, this study selected three as the weighting coefficient β value (Fig. 5A, B), making the correlation coefficient between the logarithm of the node connectivity (log (k)) and the logarithm of the probability of the node log ((p (k))) greater than 0.9. The number of genes in each gene module was more than 30, and three modules were identified by the dynamic shearing method (Fig. 5C). Moreover, to research the relation of WGCNA modules and clinical traits, this study calculated the corresponding *p*‐values and correlation coefficients among groups, age, CADi or sex and eigengenes of each module. The results showed that the three modules were significantly associated with the group traits. Turquoise module was significantly relevant to CADi and age, indicating that these lipid metabolism related genes in the modules may be more highly expressed in older CAD cases and lead to more severe CAD.

### Screening of DEG based on genes the turquoise module

**Fig. 6:**
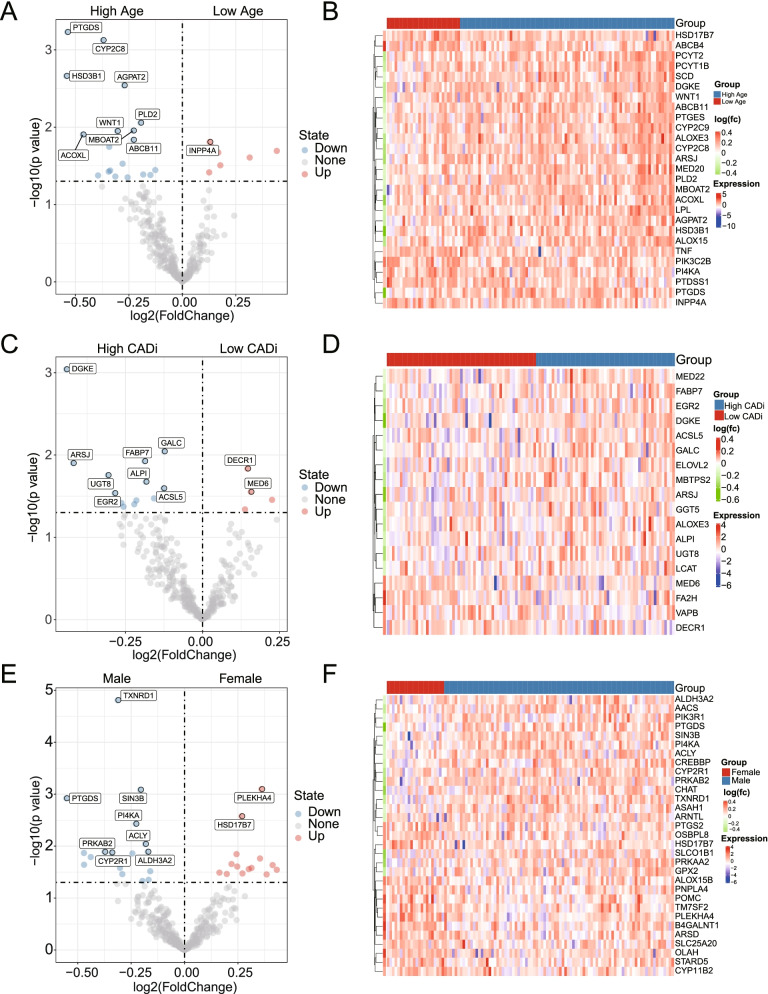
Volcano plots and heatmaps of differentially expressed genes (DEGs). DEGs are shown in volcano plots and heatmaps based on the subgroups of low- and high-age (**A**, **B**), low- and high-CADi (**C**, **D**) and male and female (**E**, **F**) CAD patients

According to age, cases were divided into two groups (low age group: < 50 years; high age group: ≥ 50 years). Genes, such as PDGTS, HSDB3D1 and CYP2C8 were highly expressed in the high age groups (Fig. 6A, B). Taking CADi = 48 as the cut-off value (Fig. 6C, D), the patients were divided into a low CADi group (CADi < 48) and a high CADi group (CADi ≥ 48). Thirteen genes were upregulated in the high CADi group, DGKE was the most significant, and 4 genes were upregulated in the low CADi group. Finally, as shown in Fig. 6E, F, 16 genes, including TXNRT1 and PDGTS, were highly upregulated in the male group. Moreover, PLEKHA4, HSD17B7 and so on were upregulated in the female group.

## Discussion

In the current study, the batch effect from different datasets were successfully removed, and this research investigated the expression of 581 lipid metabolism genes in normal controls and CAD cases from three GEO datasets. Moreover, to identify CAD subgroups associated with lipid metabolic prognosis and processes, for the first time, this research divided 352 CAD patients into three groups (NMF1, NMF2 and NMF3) based on the lipid metabolism genes screened from previous publications. The results indicate that cases in subgroup NMF2 had more severe CAD. In addition, As shown by WGCNA, the turquoise module plays an important role in CAD. In general, this study investigated the lipid metabolic landscape of CAD and found that some lipid metabolism related genes (PDGTS, DGKE and so on) were significantly related to clinical characteristics.

At present, there are many theories about the occurrence of coronary atherosclerosis, including the lipid infiltration theory, smooth muscle cell cloning theory, thrombosis theory, and endothelial injury response theory [25, 26]. The endothelial injury reaction indicates that the lipid peroxidation stress reaction and inflammatory reaction induced by various risk factors cause damage to the structure of endothelial cells, which leads to an inflammatory-fibroproliferative reaction and damages the function of endothelial cells [27]. Coronary atherosclerosis is not only a chronic inflammatory injury process but also a lipid accumulation and lipid peroxidation stress process in which a large number of lipid peroxidation stress factors are involved [28]. Therefore, lipid metabolism disorders play an important role in endothelial cell injury, so it is necessary and important to investigate the relationship between lipid metabolism genes and CAD.

According to the expression of lipid metabolism-related genes, CAD cases were further divided into three subgroups, and cases in distant subgroups showed different clinical characteristics and gene expression profiles. For instance, subjects in subgroup NMF2 tended to have an increased severity of CAD. DEGs of NMF2 were mainly enriched in regulation exocytosis, haemostatic, inflammatory mediators and vesicle-mediated transport. In CAD, the production of thrombin and lipid mediators leads to exocytosis of Weibel-Palade bodies, causing recruitment of platelets and leukocytes and fibrin deposition, which can induce further exocytosis of endothelial cells [29, 30]. Researching the mechanism of endocytosis in endothelial cells may help us to understand the recruitment of platelets and leukocytes and thrombosis. In addition, extracellular vesicles released from platelets, erythrocytes, endothelial cells and leukocytes transport much biological information to change the pathophysiological processes of CAD [31]. Moreover, the CADi and age of the NMF1 group were similar to those of the NMF3 group, but they showed a great deal of difference in their intrinsic biological characteristics. Unlike the genes of the NMF1 group, which are mainly involved in the regulation of transport, those in the NMF3 group are highly expressed in terms of the immune system. A previous study reported that individuals infected with human immunodeficiency virus (HIV) are twice as likely to have an acute myocardial infarction and stroke as people without HIV infection [32]. Apart from traditional risk factors, traits associated with HIV infection, including low CD4 + T-cell count, inflammatory response associated with HIV infection and some antiretroviral therapies, are independently related to cardiovascular disease [33]. The pathogenesis of HIV infection complicated with CAD is very complex and poorly understood, which is probably the result of the interaction of these traditional and nontraditional cardiovascular risk factors through different links in the process of chronic infection. Nevertheless, further study is needed to determine the exact mechanisms underlying HIV-related CAD.

To comprehensively investigate the relationship between clinical characteristics and lipid metabolism genes in the turquoise module. DEG screening was performed. The PTGDS expression level increased in both the older and male groups. PTGDS catalyses the conversion of prostaglandin H2 to prostaglandin D2, which is an effective platelet aggregation inhibitor [34]. PTGDS can restrain vascular smooth muscle cell proliferation and migration [35, 36]. Rezaee et al. reported that PTGDS overexpression is thought to be a negative compensatory reaction to the inflammatory events elevated by prostaglandins because of its anti-inflammatory properties [37]. In addition, the changes in the expression levels of PTGDS are opposite to the expression levels of miR-520 [37]. Therefore, this research suggested that PTGDS is a circulating marker for cardiovascular injuries and the severity of CAD. Moreover, DGKE was upregulated in the high CADi group, and DGKE had high selectivity for diacylglycerol (DAG) containing arachidonic acid and may terminate signals transmitted by arachidonoyl-DAG or contribute to the synthesis of phospholipids with specific fatty acid compositions. DGKE potentially alters LDL-C metabolism through its effects on DAG levels [38]. However, few studies are available on the function of DGKE, further research and experimental confirmations are needed to verify these findings.

### Comparisons with other studies and what does the current work add to the existing knowledge

There are two new findings from the current study: a) a new CAD classification was established based on the gene expression profile of lipid metabolism genes. and different CAD subgroups have their own intrinsic biological and clinical characteristics; b) this study found that some lipid metabolism related genes (PDGTS, DGKE and so on) were related significantly with clinical characteristics.

### Study strengths and limitations

This research applied nonnegative matrix factorization to reveal molecular subgroups in CAD based on lipid metabolic genes and furthers the understanding of the genetic diversity of CAD. This study, however, had some limitations. First, this research only focused on several major enrichment results, possibly neglecting potential genes related to CAD. Second, this study lacked experimental verification. Further experiments are needed to validate these findings.

## Conclusion

In conclusion, cases of CAD were classified from the lipid metabolic perspective, and different subgroups may have their own intrinsic biological characteristics, the classification may contribute to predicting the progress and prognosis of CAD cases and personalized therapies. Furthermore, PTGDS and DGKE may have crucial roles in the progression of CAD atherosclerosis. In addition, PTGDS may be a circulating marker for cardiovascular injuries and the severity of CAD. The findings of this study can provide new insights into CAD therapy and contribute to further understanding of its molecular mechanism.

## Data Availability

The data analysed in this study can be fetched from the GEO (https://www.ncbi.nlm.nih.gov/geo/) website.

## Abbreviations

CAD: Coronary artery disease
GO: Gene Ontology
NMF: Non-negative matrix factorization
GWAS: Genome‐wide association studies
LMRGs: Lipid metabolism related genes
CADi: Coronary artery disease index
KEGG: Kyoto Encyclopedia of Genes and Genomes
PCA: Principal component analysis
STRING: Search Tool for the Retrieval of Interacting Genes
DAG: Diacylglycerol
PPI: Protein–protein interaction
WGCNA: Weighed gene coexpression network analysis
